# Improving the Fertigation of Soilless Urban Vertical Agriculture Through the Combination of Struvite and Rhizobia Inoculation in *Phaseolus vulgaris*

**DOI:** 10.3389/fpls.2021.649304

**Published:** 2021-05-25

**Authors:** Verónica Arcas-Pilz, Felipe Parada, Gara Villalba, Martí Rufí-Salis, Antoni Rosell-Melé, Xavier Gabarrell Durany

**Affiliations:** ^1^Sostenipra Research Group (2017 SGR 1683), Institut de Ciència i Tecnologia Ambientals (CEX2019-000940-M), Universitat Autònoma de Barcelona, Barcelona, Spain; ^2^Department of Chemical, Biological and Environmental Engineering, Universitat Autònoma de Barcelona, Barcelona, Spain; ^3^Institut de Ciència i Tecnologia Ambientals, Universitat Autònoma de Barcelona, Barcelona, Spain; ^4^Institució Catalana de Recerca i Estudis Avançats, Barcelona, Spain

**Keywords:** fertigation control, struvite, rhizobium, plant physiology, nutrient uptake, soilless agricultural system

## Abstract

Soilless crop production is a viable way to promote vertical agriculture in urban areas, but it relies extensively on the use of mineral fertilizer. Thus, the benefits of fresher, local food and avoiding the transportation and packaging associated with food import could be counteracted by an increase in nutrient-rich wastewater, which could contribute to freshwater and marine eutrophication. The present study aimed to explore the use of mineral fertilizer substitutes in soilless agriculture. *Phaseolus vulgaris* (common bean) was fertilized with a combination of slow-releasing fertilizer struvite (a source of N, P, and Mg), which is a byproduct of wastewater treatment plants, and inoculation with Rhizobium (a N_2_-fixing soil bacteria). The experiment included three bean-production lines: (A) 2 g/plant of struvite and rhizobial inoculation; (B) 5 g/plant of struvite and rhizobial inoculation, both irrigated with a Mg-, P-, and N-free nutrient solution; and (C) a control treatment that consisted of irrigation with a full nutrient solution and no inoculation. Plant growth, development, yields, and nutrient contents were determined at 35, 62, and 84 days after transplanting as well as biological N_2_ fixation, which was determined using the ^15^N natural abundance method. Treatments A and B resulted in lower total yields per plant than the control C treatment (e.g., 59.35 ± 26.4 g plant^–1^ for A, 74.2 ± 23.0 g plant^–1^ for B, and 147.71 ± 45.3 g plant^–1^ for C). For A and B, the nodulation and N_2_ fixation capacities appeared to increase with the amount of initially available struvite, but, over time, deficient levels of Mg were reached as well as nearly deficient levels of P, which could explain the lower yields. Nevertheless, we conclude that the combination of struvite and N_2_-fixing bacteria covered the N needs of plants throughout the growth cycle. However, further studies are needed to determine the optimal struvite quantities for vertical agriculture systems that can meet the P and Mg requirements throughout the lifetime of the plants.

## Introduction

From 1950 to 2018, the population living in urban areas grew more than fourfold to an estimated 4.2 billion people. This unprecedented population increase has greatly increased global food demand, which has exerted great pressure on natural resources (United Nations, 2019). In response, new ways to efficiently produce vegetables while minimizing land use are being explored (Sanyé-Mengual et al., 2015, 2018). One of these initiatives is vertical farming with the use of soilless production systems with growing media or substrates (Sonneveld and Voogt, 2009), which would reduce the transportation and packaging of foodstuffs to cities (Sanyé et al., 2012). However, vertical agriculture relies extensively on the use of mineral fertilizer, which results in nitrates and phosphate being discharged into wastewater, which can contribute to freshwater and marine eutrophication (Anton et al., 2005; Gopalakrishnan et al., 2015; Sanjuan-Delmás et al., 2018).

This extensive use of mineral fertilizers affects not only the environment but can also be related to a high cost of production and extraction, as is the case for nitrogen fertilizers due to the Haber-Bosch process (Cherkasov et al., 2015) and for phosphorous due to phosphate rock extraction (Cordell and White, 2013). The widespread use of these nutrients has caused vertical farming to rely entirely on them, which thus makes this agricultural practice unsustainable in the long run. The high energy cost of synthetic nitrogen production and the ever-depleting sources of phosphate rock, when added to the environmental cost of their disposal and emissions to water and air (Rufí-Salís et al., 2020a, b), necessitates the search for alternatives to further implement these technologies in a sustainable way.

Many strategies have been described in recent years for the implementation of organic fertilization in vertical farming, which embraces a circular economy framework to reduce new resource inputs into cities. Some examples include fertilization that is based on gray water and urine (Ikeda and Tan, 1998; Karak and Bhattacharyya, 2011) and the use of biofertilizers such as Rhizobium for the cultivation of legumes (Kontopoulou et al., 2015; Savvas et al., 2018) for the plant nitrogen supply. Other methods describe the use of sewage sludge (Frossard et al., 1996), sewage sludge ash (Nanzer et al., 2014), and struvite (Rech et al., 2018) as alternative P sources. While these strategies may reduce the direct inputs of specific inorganic fertilizers, their use often results in lower crop yields and, in some cases, require more infrastructure for irrigation systems. These studies tend to focus on one particular nutrient alternative and do not consider the combination of alternative methodologies. Therefore, innovation to provide a solution for multiple mineral fertilizers while avoiding the addition of infrastructure as well as further environmental burdens due to local nutrient sourcing has not been widely studied.

Struvite (MgNH_4_PO_4_.6H_2_O), which is a crystalline byproduct of wastewater treatment plants that formes by spontaneous or induced precipitation, usually contains high N and P concentrations (Rahman et al., 2014) and is regarded as a viable slow-releasing fertilizer due to its high P, Mg, and N contents, which average 12.5%, 9.9%, and 5.7%, respectively (Ahmed et al., 2018) and are suitable for plant growth (Degryse et al., 2017; Ahmed et al., 2018). Due to struvite’s high nutrient concentrations, there are many ongoing efforts to optimize induced precipitation to make wastewater a valuable resource for providing a P alternative to the use of the depleting phosphate rock (Massey et al., 2007; Cordell et al., 2009; Talboys et al., 2016; Degryse et al., 2017).

A further positive aspect of struvite as an agricultural fertilizer substitute is its slow solubility in granular form (Talboys et al., 2016) under alkaline and neutral pH soil conditions (Bhuiyan et al., 2007). Thus, the risks of nutrient leaching and water eutrophication are rather small under these conditions when struvite is compared to common readily soluble fertilizers (Ahmed et al., 2018). Furthermore, the removal of approximately 30–40% of N and P from wastewater to produce this substance can prevent eutrophication in urban water cycles (González Ponce et al., 2009; Antonini et al., 2012). The granular form of struvite also causes it to be easily manageable and could be applied in larger-scale productions by mixing it with soil or applying it to the substrates in hydroponic production systems. The use of struvite has already been tested in agriculture as a substitute for phosphate from other sources and has shown promising results with low or even no yield losses reported (González Ponce et al., 2009; Cabeza et al., 2011; Liu et al., 2011; Ackerman et al., 2013; Degryse et al., 2017; Ahmed et al., 2018).

Although struvite already contains N that is available to plants, legumes have high N demands (McKey, 1994). Therefore, the average N contents in struvite would not be sufficient for soilless crops to achieve commercial yields and would require a second source of N to do so. This N could be obtained from Rhizobium, which is capable of forming an endosymbiotic interaction with leguminous plants by entering root cells and forming nodules. These nodules enable atmospheric N_2_ fixation and ammonia (NH_3_) formation. Plants benefit from the bacteria that generate these compounds, while the bacteria can profit from photosynthesis-derived compounds (Long, 1989). This symbiosis, on the other hand, may entail a major requirement of nutrients from the plant, such as phosphorous, to satisfy the needs of the bacteria and successful nodulation (Olivera et al., 2004). Possible N_2_ fixation depends on successful rhizobial root colonization, which is influenced by diverse factors, such as phosphorous fertilization, salinity, drought, and initial N availability (Araújo et al., 2007; Ntatsi et al., 2018; Savvas et al., 2018).

Rhizobium as a second source of N was chosen due to the lower inputs needed to achieve nitrogen intake by plants (Gopalakrishnan et al., 2015). When using the N_2_-fixing bacterium, Rhizobium, in hydroponic cultivation, Kontopoulou et al. (2017) described the need to apply initial N fertilization until nodulation in the root medium occurs, to further encourage nodulation and therefore N fixation, plant growth, and production. Even though previous studies have reported lower production capacities for N_2_-fixing plants than for common beans with N fertilization (Olivera et al., 2004; Kontopoulou et al., 2017), a combination of the two N sources (e.g., struvite and N_2_-fixing bacteria) was used to determine the possibility of overcoming such lower yields (Pampana et al., 2017; Savvas et al., 2018).

To determine how effective the two alternative fertilizers are in providing N to plants, the ^15^N natural abundance method was employed to determine the source of N throughout the experiment (Shearer and Kohl, 1989). While plants with N that is acquired from symbiotic atmospheric N_2_ fixation show the lower richness of the ^15^N isotope, which corresponds to the atmospheric abundance (0.3663%), plant tissues that are subjected to other N sources can exhibit greater amounts of the ^15^N isotope, which depend on the N fertilizer applied (Robinson, 2001).

The present study aimed to add to this growing pool of knowledge on vertical urban agriculture by exploring the use of mineral fertilizer substitutes struvite and rhizobium combined in an effort to reduce emissions of simultaneously N and P to the environment in urban vertical agriculture. This combination also aims to optimize crop yields while avoiding the installation of additional infrastructure. In this study, we analyzed the growth, development, and production of the common bean (*Phaseolus vulgaris*), which was fertilized with a combination of the slow-releasing fertilizer, struvite, and the soil bacteria, Rhizobium. A combination of these alternative fertilizers can be implemented easily in terms of cost and space and promotes nutrient recycling within cities.

## Materials and Methods

### Experimental Site, Materials, and Growth Conditions

This experiment was conducted in the Rooftop Greenhouse Laboratory (RTG-Lab) of the Environmental Science and Technology Building (ICTA-UAB), which is located in the Universitat Autònoma de Barcelona Campus (42°29′24″ E, 45°94′36″ N) (Sanjuan-Delmás et al., 2018). The bean variety used in this experiment was *Phaseolus v. Pongo*, which had previously been germinated in a commercial greenhouse 10 days before transplanting in the RTG-Lab. The production system was soilless with a perlite substrate in 40 L bags and the use of fertigation through a 2 L/h drip irrigation system.

Bean seeds were treated with a commercial product (e.g., Nadicom GmbH©) which contained a mixture of *Rhizobium phaseoli* and *Rhizobium giardinii* strains for inoculation before planting. The inoculation procedure was an exposure of the plant seeds with the liquid commercial product before planting. A total of 5 days after the seedling was transplanted into the perlite substrate, 5 ml liquid commercial mix was added to each plant, therefore ensuring the presence of the bacteria in the substrate. Once the plants were inoculated, they were irrigated with an Mg-, P-, and N-free solution (Supplementary Table 1b), and application of K_2_SO_4_ was increased to adjust for the K requirements. The control plants, on the other hand, were irrigated with a full nutrient solution. These nutrient concentrations were maintained throughout the entire experiment. The crops were irrigated four times a day for 3 min, which provided a total amount of 400 ml per day per plant.

The inoculated plants were treated with two different struvite amounts placed inside the perlite bag around the root area and surface, varying the concentration of P and N available to the plant from struvite: (A) 2 g (1.02 mmol of P and 0.46 mmol of N) of granulated struvite per plant and (B) 5 g (2.57 mmol of P and 1.15 mmol of N) of granulated struvite per plant. The amount of struvite that was best for growth was determined in a previous experiment conducted in the same i-RTG in which 2.57 mmol P was deemed sufficient for common bean fertilization to reach an equivalent level of commercial production as that of mineral-fertilized beans. To ensure no struvite loss due to runoff, each plant was planted inside an additional 1 L bag containing perlite and the corresponding amount of struvite, with small holes to allow water drainage.

Each treatment was arranged randomly in four rows with 16 plants each (four perlite bags with 4 plants per bag were planted in a frame with an area of 0.125 m^2^), which resulted in a total of 64 plants per treatment (e.g., A, B, and Control), with 192 plants in total (Supplementary Figure 1b). Due to the irrigation and leachate recovery systems, randomization could only be achieved for entire lines of four bags.

The plants were germinated and transplanted in duplicate and were thinned to one plant at 21 days after transplanting (DAT).

Greenhouse conditions were monitored during the entire experiment with T107 sensing devices (Campbell Scientific) that were placed along the cropping area to measure temperature, relative humidity, and radiation (see Supplementary Table 2b). To ensure proper plant irrigation drainage volumes, the pH and electrical conductivity levels of the leachate were recorded every day for each irrigation line.

The phenological stages of the bean plants were determined each week. This information was assessed to identify plant growth, development, and productivity over time and provided a clear view of the plant cycle, growth, and production peaks that enabled accurate comparisons of plant development between treatments and the control. This was performed by counting leaves, flower buttons, and open flowers. The number of ripened bean pods was also counted and weighed for each harvest. These measurements were performed for each of the eight plants that were in the two middle bags of each row (see Supplementary Figure 1b) and started 14 DAT. To ensure uniform counting, leaves under 5 cm length were not considered, and only fully formed flower buttons with white coloration and fully open flowers were counted. For the bean pods, a minimum length of 11 cm was used for harvesting, while bean pods shorter than this were retained for the next harvest. The average numbers and bean pod weights per treatment were then calculated for each week. At the same time and on a weekly basis, chlorophyll content measurements were performed (with a SPAD CCM-200 plus; Opti-Sciences, Inc.) on the same eight plants in the center of each row.

### Description of Plant Sampling Methods

To determine the changes in plant development as well as nutritional states and ^15^N concentrations, samples were taken during the three different crop stages. The first sampling took place 35 DAT, immediately before bean pod production started; the second sampling took place 62 DAT, during the productive phase of the plants; and the last sampling took place 84 DAT, at the end of the productive stage, which marked the last day of the experiment.

The samples consisted of eight randomly chosen plants per treatment (excluding the eight central plants of each row that were kept for phenological analysis). Each plant was washed with deionized water, excess water was dried off and each plant was separated into the four main organs: leaves, shoots, roots, and nodules. These were then weighed separately to determine their fresh weights (FW). All organs were placed separately in envelopes and left to dry in an oven at 65°C until stabilized dry weights (DW) were obtained, which occurred after approximately 7–8 days. The means of the obtained values were calculated for each treatment, each organ, and time. The numbers of nodules were counted prior to drying to determine the mean nodulation of each plant. In addition, fruit samples from each treatment were taken at three different times (49, 62, and 77 DAT), which closely matched the three plant harvests.

Moreover, 25% of the total sampled leaves for each plant were separated to determine their areas before the drying process. To do so, these fresh leaves were scanned with a reference pixel to obtain leaf areas using ImageJ software (Rueden et al., 2017). These leaf areas were further extrapolated to 100% of the leaf biomass of the plant. The leaf area index (LAI) was then calculated by dividing the total leaf area by the area of the planting frame of our crop (0.125m^2^).

### Nitrogen Isotopic (δ^15^N) Analysis

The goal of inoculating treatments A and B with Rhizobium was for the plants to indirectly fix N_2_ from the air and meet their N needs in this way. To determine how much of the N assimilated by the plants came from the atmosphere, we used the natural abundance method (Shearer and Kohl, 1989) to identify the origin of the N that was obtained by the plants, which in our case, should be either struvite or atmospheric N. While treatments A and B were actively inoculated with Rhizobium strains and fertilized with struvite containing N, the control treatment was fertilized through standard N fertilization that was administered through irrigation. Additional nitrogen sources were not considered due to the laboratory conditions and production of inert perlite.

Analysis was performed with an elemental analyzer isotopic ratio mass spectrometer (EA-IRMS; Thermo Fisher Scientific). The devices used were a Flash EA 1112 analyzer and Delta V Advantage spectrometer that was coupled with a Conflo III interface. The plant and struvite samples were weighed in tin capsules and were introduced into the EA-IRMS system to obtain the δ^15^N values, as calculated with the following equation (Eq. 1) (Robinson, 2001):

$$\delta^{15}\,N=\frac{S\,a\,m\,p\,l\,e\,a\,t\,o\,{m\%}^{15}\,N-0.3663}{0.3663}\times 1000$$

*Equation 1: δ^15^N is the natural tracer for our N sources, the sample atom %15N is the previously obtained value of our plant sample, and the value 0.3663 is a standard value that represents the percentage of ^15^N in the atmosphere.*

δ^15^N values provide an indication of the N sources in plant tissues. Values close to 0 indicate that the plant N sources are mainly due to atmospheric N_2_ fixation, while higher values can be interpreted as indicating mixed sources or those dominated by the N obtained from struvite. The δ^15^N value obtained for the struvite used in the experiment was 7.1‰. To determine the relative contributions from the two sources considered, we used Eq. 2, which yields an estimate of the percentage of N that was derived from N_2_- fixation (%Ndfa) (Shearer and Kohl, 1993; Unkovich et al., 2002; Arndt et al., 2004)

%Ndfa=δ15⁢N⁢S⁢o⁢u⁢r⁢c⁢e⁢ 2-δ15⁢N⁢S⁢i⁢n⁢kδ15⁢N⁢S⁢o⁢u⁢r⁢c⁢e⁢ 2-′B′⁢v⁢a⁢l⁢u⁢e×100

*Equation 2: %Ndfa (nitrogen derived from N_2_ fixation from the atmosphere), δ15N Source 2 (‰) corresponds to the δ15N value of struvite, δ15N Sink (‰) corresponds to the δ15N value from the sample, and the “B” value corresponds to the δ15N of N_2_ fixation taking into account possible fractionation.*

The “B” value is the isotopic fractionation observed in N_2_-fixing *P. vulgaris* was set to –1.16‰, which corresponded to the lowest δ^15^N value obtained (Shearer and Kohl, 1989; Peoples et al., 2002; Kermah et al., 2018) and was similar to the values determined by Kontopoulou et al. (2017) in common bean that was fertilized without N and inoculated with Rhizobium.

The biologically fixed nitrogen (BNF) levels were further calculated with the obtained %Ndfa values as well as the obtained values for the nitrogen contents in the plants. To extrapolate to kg/ha, a theoretical plant density of eight plants/m^2^ was used.

Finally, the nitrogen use efficiency (NUE) for all treatments was estimated. The methodology that was followed to perform these calculations was given by Weih (2014), who provided a tool to successfully calculate the NUE. To accomplish this, the information provided was as follows:

– N content at the initial stage of the plant in g/m^2^ (previous to the main production stage at 35 DAT),
– N content at the main productive stage in g/m^2^ (chosen at 84 DAT),
– N content in the harvested yield in g/m^2^,
– Biomass yield g/m^2^,
– Added N to the soil in g/m^2^ (in this case, perlite).

### Plant Nutritional Analysis

Dried and ground plant organs were weighed (up to 0.25 g) and digested using a single reaction chamber microwave (Milestone Ultrawave) with concentrated HNO_3_. The digested samples were then diluted with 1% HNO_3_ (v/v) and were analyzed by optical spectrometry (ICP-OES) (Perkin-Elmer, Optima 4300DV). All samples were weighed, digested, and analyzed in duplicate.

### Statistical Analysis

All statistical analyses in this experiment were performed with R studio software. Data normality in our values was tested with Shapiro-Wilk test *p* > 0.05, and to ensure homogeneity of variance the Levene test was performed *p* > 0.05. When both criteria were validated Duncan’s multiple range test was used to assess the statistical significance of treatments. The Kruskal-Wallis test was used for no parametric data. The significance between the treatments was tested for each harvest time separately.

## Results

### Phenology, Biomass, and Yield

**FIGURE 1:**
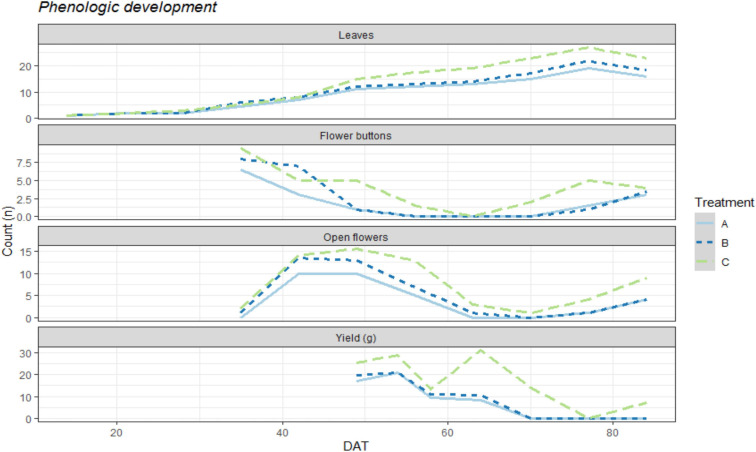
Graphic representation of the mean numerical count per plant for each organ (Leaves, flower buttons, open flowers) and yield in g/plant on a weekly basis, DAT representing the days after transplanting inside the iRTG. The colors represent the three treatments: A = N.free solution with Rhizobium inoculation and 2g of struvite per plant. B = N.free solution with Rhizobium inoculation and 5g of struvite per plant. C = Complete nutrient solution without struvite and no inoculation treatment.

Weekly recordings of the phenological growth of the bean plants exhibited differences among all treatments (Figure 1). In this figure, we can see the evolution throughout the crop development of biomass production as well as flower production and finally bean pod production. The control plants (Treatment C) showed greater biomass growth and faster development in their transitions from flower buttons to open flowers and bean pod production. Although the treatment performances were similar in the earlier growth stages, once the production stage started, greater differences were observed.

At 40 DAT and 50 DAT, treatments A and B began to perform worse for leaf production as well as for the formation and opening of flower buttons than the control plants (C). Between 60 DAT and 70 DAT, a second production peak can be seen for the control treatment as well as rapid generation of flower buttons, while treatments A and B showed a declining pattern for bean pod production.

Table 1 shows the changes in the plant measurement results that were conducted on the sampled plants at three different developmental stages. While the first period of plant sampling, 35 DAT, showed very few significant differences among the treatments (only in the case of dry weight), the later samplings at 62 and 84 DAT showed greater differences between treatments. At this point, the leaf and shoot dry weights were greater for the control treatment, as was the measured leaf area index. The only parameter without significant differences among treatments throughout the entire experiment was the root dry weight at 62 DAT. The dry weights of the nodules exhibited persistent, significant differences for the three samplings among treatments A and B and control treatment C and reached maximum values of 0.16 g, 0.12 g, and 0.05 g for treatments A, B, and C, respectively. On the other hand, treatment B (with higher struvite quantities) also exhibited significantly greater numbers of nodules as well as higher weights than the other two treatments during the third sample period.

**TABLE 1:** Results for the mean values (*n* = 8) per plant of fresh weight (FW) and dry weight (DW) of the different organs as well as the Leaf Area Index (LAI) m^2^ plant^–1^ of the three treatments (A = 2g Struvite + Rhizobium; B = 5g Struvite + Rhizobium; C = Control) in three different time periods: 35 DAT (1), 62 DAT (2), and 84 DAT (3).

	Leaf DW (g) per plant	Shoot DW (g) per plant	Roots DW (g) per plant	Nodules n per plant	Nodules DW per plant (g)	LAI
**(1)**						
A	1.12^a^ ± 0.22	0.46^a^ ± 0.08	0.44^a^ ± 0.10	132.50^a^ ± 80.35	0.16^b^ ± 0.07	0.57^a^ ± 0.12
B	1.31^a^ ± 0.46	0.56^a^ ± 0.19	0.51^a^ ± 0.15	156.75^a^ ± 60.82	0.12^b^ ± 0.06	0.62^a^ ± 0.23
C	1.33^a^ ± 0.57	0.58^a^ ± 0.18	0.53^a^ ± 0.14	148.75^a^ ± 48.23	0.05^a^ ± 0.02	0.65^a^ ± 0.27
**(2)**						
A	3.97^a^ ± 1.25	2.02^a^ ± 0.72	0.80^a^ ± 0.28	127.88^a^ ± 63.85	0.14^b^ ± 0.09	1.28^a^ ± 0.51
B	3.69^a^ ± 1.53	2.24^a^ ± 1.01	0.87^a^ ± 0.34	172.25^a^ ± 132.66	0.15^b^ ± 0.14	1.29^a^ ± 0.64
C	6.44^b^ ± 3.09	3.85^b^ ± 1.95	0.95^a^ ± 0.44	82.25^a^ ± 62.47	0.01^a^ ± 0.01	2.64^b^ ± 1.33
**(3)**						
A	5.86^a^ ± 2.96	3.09^a^ ± 1.45	1.77^a^ ± 0.79	136.88^b^ ± 106.31	0.15^b^ ± 0.13	1.74^a^ ± 0.92
B	7.40^a^ ± 2.17	4.53^a^ ± 1.48	2.49^a^ ± 0.57	186.25^c^ ± 48.79	0.24^b^ ± 0.11	1.80^a^ ± 0.67
C	11.11^b^ ± 1.51	6.91^b^ ± 1.42	3.35^b^ ± 0.88	39.13^a^ ± 24.76	0.02^a^ ± 0.02	3.72^b^ ± 0.87

*Significant differences (*p* < 0.05) between treatments marked with different letter (a,b,c).*

When examining the SPAD measurements (Supplementary Figure 2b), some differences in chlorophyll content were observed throughout the experiment. Initially, we can see a significant difference between the A and B treatments and the control marking a greater chlorophyll content in the latter that is sustained until 35 DAT. From 42 DAT to 63 DAT, the chlorophyll content in treatments A and B increases while treatment C remains stable. While differences toward the end of the experiment remain small, we can appreciate a greater chlorophyll content in the struvite fertilized treatments.

The final production amounts that were obtained for all three treatments were 1899.2 g, 2375.6 g, and 4726.7 g of green bean pods for treatments A, B, and C, respectively. Although the plants treated with struvite and rhizobium produced approximately half the yield of the mineral-fertilized plants, it is important to note that they were healthy throughout the experiment. The average yields provided per plant were 59.35 ± 26.4 g^a^ plant^–1^ for A, 74.24 ± 23.0 g^a^ plant^–1^ for B, and 147.71 ± 45.3 g^b^ plant^–1^ for the control treatment C. These production differences can also be seen in Figure 1 where the obtained yields are shown as a function of time and show greater production peaks and a more rapid ability to develop flower buttons and open flowers after each harvest.

### δ^15^N, %Ndfa and Biologically Fixed N

**FIGURE 2:**
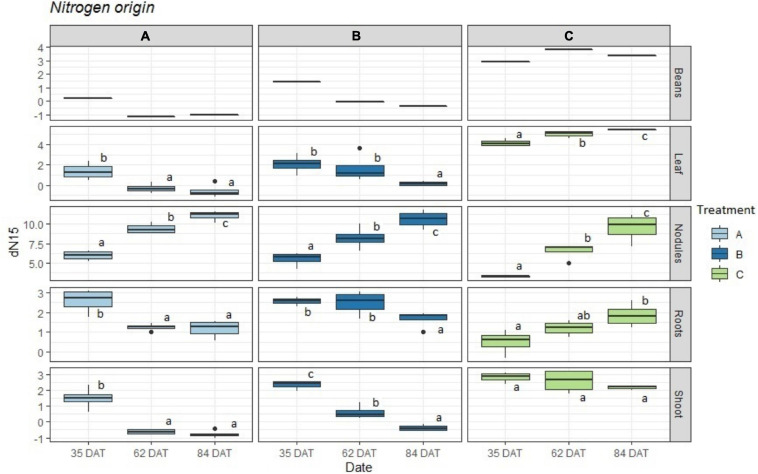
Boxplot representing the obtained δ^15^N values (*n* = 4) for treatments: A = 2g of struvite + Rhizobium inoculation + P, Mg, N-free nutrient solution, B = 5g of struvite + Rhizobium inoculation + P, Mg, N-free nutrient solution and C = standard nutrient solution - Rhizobium inoculation. These observed values are given by plant organs in three different time periods: 35 days after transplanting, 62 days after transplanting, and 84 days after transplanting. Significant differences (*p* < 0.05) between dates marked with different letter (a,b,c).

The results obtained for the δ^15^N values of plant tissues and bean pods as seen in Figure 2 (and Supplementary Figure 3b) show great variability in the enrichment of all organs except for the nodules. While treatment C showed clear enrichment over time, the pattern for treatments A and B was the opposite. For the nodules, all three treatments exhibited clear enrichment over time. Treatment B exhibited intermediate δ^15^N values that were between those of A and C, with decreasing δ^15^N values that were not as abrupt when compared to the tissues that were exposed to treatment A. It was also interesting to observe that the major decrease in δ^15^N values for treatment A occurred between days 35 and 62 after transplanting and remained rather constant at 84 DAT. For the plants in treatment B, the value at 62 DAT did not fall as drastically and experienced a more significant change at 84 DAT.

**FIGURE 3:**
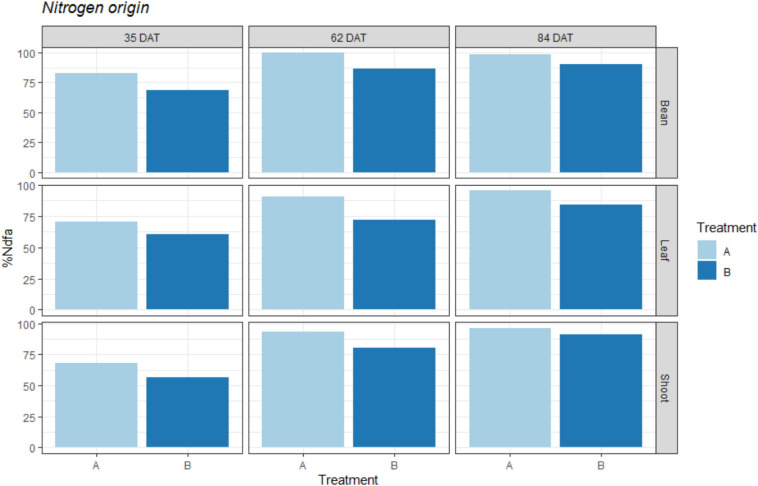
Percentage of Nitrogen derived from atmospheric N2 fixation (%Ndfa) represented for treatments: A = 2g of struvite + Rhizobium inoculation + P, Mg, N-free nutrient solution, and B = 5g of struvite + Rhizobium inoculation + P, Mg, N-free nutrient solution. These observed values are given for two plant organs (leaf; shoot) as well as the bean pods in three different time periods: 35 days after transplanting, 62 days after transplanting, and 84 days after transplanting.

When calculating the percentage of fixed atmospheric N during our three sampling periods, we obtained the values shown in Figure 3. This figure shows the approximate percentages of N that were derived from atmospheric fixation relative to the total N obtained by the plants.

As shown in the figure, the percentages of fixed N_2_ in all three tissues were higher for the plants in treatment A, with values of 65-80% at 35 DAT, which reached 90% by the end of the experiment (84 DAT). On the other hand, treatment B exhibited lower values throughout the experiment, with initial values close to 50% to 60% (35 DAT), which reached final values of 80% at 84 DAT.

While the plants with less struvite in the root medium (treatment A) increased their percentages of fixed N_2_ more rapidly (from 70% (35 DAT) to 90% (62 DAT) in the leaves), the plants in treatment B took longer to reach this value (from 60% (35 DAT) to 71% (62 DAT) in leaves). This corresponds to the results for the δ^15^N values shown in Figure 2.

Table 2 shows the results of the estimations of biological fixed nitrogen (BNF) contents expressed in kg/ha. These results show the extrapolations of total N found in the plants for each treatment to kg/ha values. The N percentages that were of atmospheric origin (obtained previously) were further used to attain the kg/ha of biologically fixed nitrogen for each treatment as well as the N from struvite that was used by the plants.

**TABLE 2:** Results for percentage of Nitrogen derived from atmospheric N_2_ fixation (%Ndfa) in plant, Total amount of N in plant expressed in kg/ha (Leaves+Shoot+Root+Beans) and Biologically fixed N expressed in kg/ha.

Date	Treatment	% Ndfa plant^–1^	Total N in plant kg/ha	Kg/ha biologically fixed N	Kg/ha N from struvite
35 DAT	*A*	68%	7.5 ± 1.0^a^	5.4 ± 1.0^a^	2.2
	*B*	60%	8.6 ± 2.2^a^	5.3 ± 1.4^a^	3.3
62 DAT	*A*	89%	24.7 ± 5.0^a^	22.9 ± 4.0^ b^	1.8
	*B*	73%	24.6 ± 6.2^a^	18.7 ± 5.1^a^	5.9
84 DAT	*A*	90%	27.3 ± 12.8^a^	25.4 ± 13.0^a^	1.9
	*B*	82%	35.0 ± 9.2^a^	29.2 ± 7.8^a^	5.8

*Results given for three treatments (*n* = 8 each) (A) 2g of struvite + Rhizobium inoculation + P, Mg, N-free nutrient solution (B) 5g of struvite + Rhizobium inoculation + P, Mg, N-free nutrient solution at three different time periods. 35 days after transplanting, 62 days after transplanting and 84 days after transplanting. Significant differences (*p* < 0.05) between treatments marked with different letter (a,b,c).*

Here, we can see that as the percentages of atmospheric-derived N and total N that were found in the plants increased, as well as the kg/ha values of biologically fixed N. While the plants in treatment A had higher values of biologically fixed N during the first two sampling periods at 84 DAT, the increase in the fixation percentage and total N in the plants in treatment B increased their amounts of biologically fixed N. On the other hand, the use of N from struvite increased only for treatment B and remained constant for treatment A.

### Nutrient Content

**FIGURE 4:**
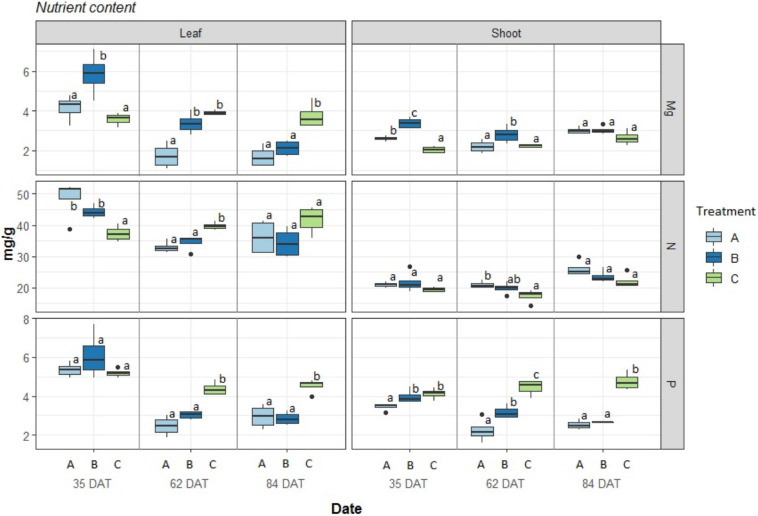
Nutrient concentration in Phaseolus vulgaris leaves and shoots, expressed in mg/g. Boxplot (*n* = 4) results given for three treatments: A = 2g of struvite + Rhizobium inoculation + P, Mg, N-free nutrient solution, B = 5g of struvite + Rhizobium inoculation + P, Mg, N-free nutrient solution and, C = standard nutrient solution - Rhizobium inoculation at three different time periods: 35 days after transplanting, 62 days after transplanting, and 84 days after transplanting. Significant differences (*p* < 0.05) between treatments marked with different letter (a,b,c).

The nutrient contents in the aboveground plant organs are presented in Figure 4 (Supplementary Figure 5b for differences between harvests). The observed concentrations of nutrients in leaves for the three treatments were at sufficient levels except for the less than optimal Mg^2+^ concentrations at 62 DAT for treatments A and at 84 DAT for treatments A and B and were close to deficient levels P in both treatments A and B at 62 and 84 DAT according to Hochmuth et al. (2018). In the case of N, in both leaf and shoot tissues, no deficient levels were found for any of the treatments, and no significant differences were found among treatments. On the other hand, a clear decline in P and Mg^2+^ over time can be seen for treatments A and B in the leaves as well as for P in the shoots. The control treatment (C), on the other hand, remained stable.

Supplementary Figure 4b also indicates the total nutrient contents that are bound to the total biomass of the sampled plants. Here, it is apparent that treatment B, with more struvite, provided results that were between those of the other treatments. In the case of Mg at 35 DAT in leaves, treatment B showed levels as high as those for the control treatment, but while the latter remained constant over time, both A and B decreased. The same trend can be seen for P in both leaves and shoots. In the case of N, we can see an increase for all treatments that was faster for control C, while A and B increased in a similar fashion.

Finally, the NUEs obtained for all three treatments were 1.32 g/g, 0.55 g/g, and 0.29 g/g for A, B, and C, respectively. The calculation methodology considered that N was in the soil, while the fixed nitrogen was not considered; therefore, the use efficiency can be very different for all three treatments.

## Discussion

### Plant Growth and Development

The results indicate that once the first production peak was reached, the control plants were more capable of continuing to produce flower buttons, while the inoculated and struvite-fertilized plants took longer. The relationship between their development and the amount of struvite given to the plants seems to be directly correlated. Generally, the biomass and bean pod production was higher in the control plants, while treatment B had a greater amount of struvite (5 g). Treatment A, with the lowest amount of struvite (2 g), was determined to be the treatment with the lowest growth and production rates. These findings agree with those presented in previous literature (Nanjareddy et al., 2014), for which lower KNO_3_ availability was directly linked to a reduction in leaf and flower formation. This reduction also seemed to be related to the P and Mg availability over time due to struvite depletion, considering that the initial performance was similar in all three treatments.

By observing the SPAD measurements, the chlorophyll contents in all three treatments indicated that the N contents in the leaves were not strongly affected by the treatments but rather the LAI. Lower P availability resulted in a reduction in LAI as well as in overall plant growth, which was observed in treatments A and B. These differences were not as great as those for root weights (compared to the other plant organs), which have been reported in the previous literature to be less affected by P reductions (Chaudhary and Fujita, 1998; Rao et al., 2008).

The lower nodule dry weights in the control treatment, compared to treatments A and B, have previously been reported in other studies, in which the nodule fresh and dry weights were found to be considerably reduced when inorganic NO_3_^–^ fertilization was not restricted (Nanjareddy et al., 2014; Kontopoulou et al., 2017). On the other hand, other authors report that the nodule number was not affected when exposing the crop to mineral and organic N sources but rather affected in size and weight (Pampana et al., 2017).

The increasing nodule numbers and weights throughout the experiment for the B treatment (with greater struvite), when compared to treatment A, confirm Kontopoulou et al. (2017). findings that low initial N fertilization can restrict successful colonization. These differences, however, could also be due to the lower P amounts in treatment A compared to treatment B since P is a limiting factor for successful nodulation (Olivera et al., 2004).

The lower bean productivities were similar to those in the study reported by Olivera et al. (2004), where bean production with lower P fertilization and Rhizobia inoculation turned out to be insufficient to reach production levels as high as those of conventionally fertilized beans. However, struvite fertilization seemed to increase the production of inoculated plants by up to 25% when treatments with 2 g and 5 g per plant were compared (59.35 ± 26.4 g plant^–1^ in treatment A and 74.23 ± 23.0 g plant^–1^ in treatment B).

The effect of the struvite treatment on the increasing nodule number and dry weight indicates successful nodulation and a greater fixation capacity with the given N. The slow release of N has presented itself as sufficient to increase the nodulation capacity as well as production capacity, without inhibiting N^2^ fixation by the bacteria.

### The Effect on Atmospheric N Fixation Capacity

The aboveground organs showed a clear pattern throughout the three measurements in terms of N assimilation. ^15^N enrichment levels in the A and B treatments were lower than that in the C treatment, which means that treatments A and B obtained most of their N from the atmosphere. This difference became even greater as time progressed and reflected a greater dependence on N_2_ fixation in the A and B treatments. The differences between these two treatments (A and B) themselves can be due to the greater availability of struvite in the root medium and therefore a greater availability of initial N and P for treatment B than for treatment A (Olivera et al., 2004; Kontopoulou et al., 2017).

The δ^15^N reductions in treatments A and B over time corresponded to the availability of N provided by the struvite, assuming that it decreased over time. These reductions can be seen when the NO_3_^–^ concentrations in the drained water were examined (see Supplementary Table 3b). While initially greater amounts of N were detected in the leached water, by the end of the experiment, very low concentrations were seen. Therefore, while the δ^15^N values for the control treatment C remained constant over time (except in the nodules), the δ^15^N values for treatments A and B decreased progressively over time, which corresponded to the available N that was provided by struvite in the root medium.

This information indicates that a change in the source of N for the plants took place during the time span of 35 to 62 DAT. We can therefore assume that the availability of struvite and therefore N in the root medium was depleted mainly during that time, which forced the plants to rely on atmospheric N_2_ fixation. The results obtained for %Ndfa also confirm that the levels of N_2_ fixation increased over time in both treatments.

The indicated timespan of 35 to 63 DAT corresponds to the initial pod production of the plant, maximizing its nutritional needs. Therefore, an N and P source capable to uphold these needs during this stage must be contemplated. As seen in Supplementary Table 3b a major reduction of NO_3_^–^ in the leachate water is found between day 35 and 49 for treatments A and B, indicating that the administered struvite was insufficient to further support a greater production.

The nodules appeared to be highly enriched with ^15^N during all three harvests, especially for treatments A and B. These results agree with previous literature that attributes this enrichment to the export of ^15^N-depleted ureides and import of ^15^N-enriched amino acids. Nevertheless, these values do not have a great effect on the total plant enrichment if the nodule biomass is considered (Shearer and Kohl, 1986; Unkovich, 2013; Craine et al., 2015).

The quantity of fixed nitrogen did not reach 40-50 kg/ha, which corresponds to low ranges, as reported in previous research (Farid and Navabi, 2015). While treatment A, with less struvite, had higher BNF values at the first two sampling times, treatment B’s BNF value had increased by the end of the experiment. These findings are in agreement with those mentioned in the literature, where BNF was found to be restricted in the presence of plant-available NO_3_^–^, and the BNF values increased during the mature stages of the plant with sufficient NO_3_^–^ fertilization during early plant growth (Müller et al., 1993; Hungria et al., 2006; Kontopoulou et al., 2017).

### Plant Health and Nitrogen Assimilation

We conclude that all treatments had sufficient N since there were minimal differences in N concentrations in the shoots and leaves during plant growth and at the end of the experiment, as was also found by Kontopoulou et al. (2015). We consider that the lower yields were caused by the reduced uptake of K^+^ and Mg^2+^ cations, which was cased by the electrochemical imbalance generated by the reduced presence of NO_3_^–^ in the root medium. This idea is reinforced by the results shown in Supplementary Figure 4b, where N gradually increased in all three treatments throughout the experiment, which indicated that fixation was taking place for treatments A and B. The values increased from less than 0.1 g N at 35 DAT up to 0.2 g at 84 DAT for both the A and B treatments.

The slight increase in K by the end of the experiment in the plants with less struvite (treatment A) was most likely due to the lower availability of the Mg^2+^ cation, which facilitated cation uptake (Marschner, 2002).

The declining N concentrations in the leachates led us to believe that the decreases in P and Mg concentrations in the aboveground organs could also be related to the depletion of struvite in the medium. This depletion occurred faster in treatment A than in treatment B, which was related to the initial amounts of struvite provided in each treatment (2 g and 5 g, respectively). It was seen that for the inoculated plants, greater amounts of P were needed to support symbiosis and nodulation, as has also been observed by other researchers (Olivera et al., 2004; Ntatsi et al., 2018; Savvas et al., 2018). Whether the additional required P can be assimilated by adding more struvite to the substrate is worth pursuing in future studies.

These findings lead to the concept that a lack of N is not the limiting factor that is entirely responsible for the lower yields of the A and B treatments, but the limiting factor is instead the progressive loss of P and Mg in the root medium as well as the reduced cation uptake. When examining the NUEs that were obtained for all treatments, it is evident that plants with lower N inputs have greater use efficiency. This difference is very clear in treatment A with a three-times higher efficiency compared to treatment B. These differences can also be influenced by atmospheric N fixation, which was not provided as “Soil” N in the calculation tool (Weih, 2014). A higher fixation capacity can therefore generate a higher NUE, which corresponds to our BNF results.

For production on larger-scale vertical farms, fertilization with struvite and Rhizobium seems possible, especially with greater struvite quantities, as in treatment B, which shows great compatibility with soil bacteria and produces larger yields than those crops fertilized with only 2 g of struvite. The initial fixation capacity of the control treatment and appearance of nodules during the first sampling stage indicate that nodulation could occur even with naturally occurring Rhizobium, which could simplify the fertilization process in soil-based agriculture. A limitation for larger-scale production could be providing precise applications of struvite in the root areas. As seen in this study, there were large production differences between the applications of 2 g and 5 g of struvite, and large-scale production in a vertical farm would mean precise weighting of the struvite amounts per plant and direct applications to each rhizosphere of each plant. As stated by Degryse et al. (2017), the location of this slow-releasing fertilizer can have a great impact on successful nutrient delivery to plants. These could thus be highly time- and resource-consuming applications.

## Conclusion

This work aimed to study the feasibility of using struvite and inoculation with Rhizobium bacteria as alternative Mg, N, and P fertilization methods for vertical agriculture systems. For this purpose, we quantified the nitrogen sources, production, and evolution of the phenological stages of *Phaseolus vulgaris* with Rhizobium inoculation and different quantities of struvite and compared the results to a control treatment. Three main conclusions can be drawn from this study.

First, both alternative fertilizer treatments supplied the necessary nutrients to fulfill the plant cycle in soilless growing media. The lower yields compared to the control suggest the necessity for evaluating higher struvite quantities to fulfill plant requirements to achieve higher yields. Since previous experiments conducted with struvite suggested successful performance with 5 g/plant, its combination with the soil bacteria, Rhizobium, causes this quantity to be insufficient due to the additional nutritional requirements of the bacteria. This can be seen by the great reduction in yields of treatments A and B in comparison to the control.

Second, while nodulation seemed to not be hindered by nitrogen input through struvite in the root medium, it did not significantly improve it either, although BNF appeared to increase in the later stages for plants grown under the treatment with a greater initial quantity of struvite.

Third, the limiting factor for struvite-fertilized and rhizobia-inoculated treatments did not seem to be nitrogen, which was maintained at sufficient concentrations in the plants throughout the experiment, but rather was potassium, due to the lower uptake capacity that was caused by an electrochemical imbalance that was generated by the reduced presence of NO_3_^–^ in the root medium as well as by magnesium and phosphorus, given that struvite depletion was reflected by the reduced plant nutrient concentrations over time.

An increase in the amount of applied struvite might be a solution for a more sustained phosphorus and magnesium supply for vertical agriculture but could also interfere with the nodulation capacity of the plants. Furthermore, we encourage the addition of nutrients in the form of anions to ensure the electrochemical balance in the root area in case NO_3_^–^ is removed. In this sense, further studies should aim to determine the optimal struvite quantities for hydroponic bean production in combination with Rhizobium inoculation.

## Data Availability Statement

The raw data supporting the conclusions of this article will be made available by the authors, without undue reservation.

## Author Contributions

VA-P contributed to the conceptualization, performed the methodology, carried out the formal analysis and data curation, and wrote the original draft. FP performed the methodology, carried out the data curation, and wrote, reviewed, and edited the manuscript. MR-S performed the methodology, wrote, reviewed, and edited the manuscript. GV contributed to the conceptualization, performed the methodology, wrote, reviewed, and edited the manuscript, supervised the data, and carried out the funding acquisition. AR-M performed the methodology and reviewed and edited the manuscript. XG contributed to the conceptualization, performed the methodology, wrote, reviewed, and edited the manuscript, supervised the data, and carried out the funding acquisition. All authors contributed to the article and approved the submitted version.

## Conflict of Interest

The authors declare that the research was conducted in the absence of any commercial or financial relationships that could be construed as a potential conflict of interest.
