# Supplementary material for: Improving the Fertigation of Soilless Urban Vertical Agriculture Through the Combination of Struvite and Rhizobia Inoculation in Phaseolus vulgaris
Source: Front Plant Sci. 2021 May 25;12:649304. doi: 10.3389/fpls.2021.649304 (PMC8186444; doi:10.3389/fpls.2021.649304)
Supplement: Supplementary file 1 [file Data_Sheet_1.docx]

***Supplementary information:***

*Table 1b: Applied nutrient solutions (NS) for the control treatment and the Mg, P, N-free nutrient solution for treatments inoculated treatments additionally fertilized with struvite.*

| Nutrients applied | Control NS | Mg, P, N-free NS | |
| --- | --- | --- | --- |
| KPO_4_H_2_ | 136 mg/L | --- |  |
| KNO_3_ | 101 mg/L | --- |  |
| K_2_SO_4_ | 217 mg/L | 435 mg/L |  |
| Ca(NO_3_)_2_ | 164 mg/ | --- |  |
| CaCl_2_ | 111 mg/L | 111 mg/L |  |
| Mg(NO_3_)_2_ | 148.3 mg/L | --- |  |
| Hortilon | - 1. mg/L | 0.1 mg/L |  |
| Sequestrene | 0.1 mg/L | 0.1 mg/L |  |

*Table 2b: Climatic conditions inside the RTG Lab.*

| *Temperature* |  |
| --- | --- |
| *Average T ºC* | *18.94* |
| *Minimum T ºC* | *4.48* |
| *Maximum T ºC* | *29.89* |
| *Standard Deviation* | *2.09* |
| *Realative Humidity* |  |
| *Average (RH)* | *38.11* |
| *Minimum (RH)* | *5.65* |
| *Maximum (RH)* | *77.37* |
| *Standard Deviation* | *5.83* |

Table 3b: Leachate NO3- content (mg/L) from results given for three treatments A) 2g of struvite + Rhizobium inoculation + P, Mg, N-free nutrient solution B) 5g of struvite + Rhizobium inoculation + P, Mg, N-free nutrient solution and C) standard nutrient solution - Rhizobium inoculation at five time periods from the 14 DAT until 77 DAT.

| **Date** | **A** | **B** | **C** |
| --- | --- | --- | --- |
| **14 DAT** | *7,71* | *10,57* | *8,54* |
| **35 DAT** | *3,41* | *4,89* | *32,89* |
| **49 DAT** | *0,92* | *0,91* | *47,87* |
| **63 DAT** | *0,03* | *0,36* | *51,97* |
| **77 DAT** | *0,32* | *n.a.* | *55,93* |


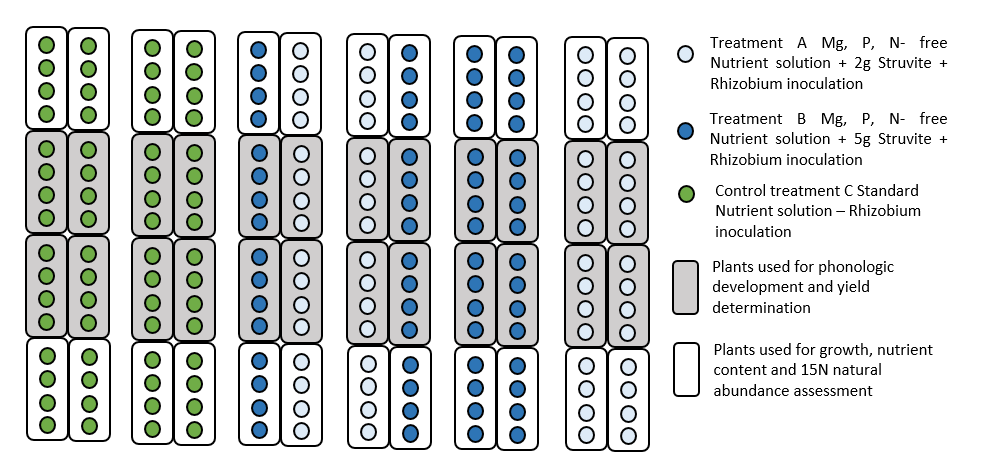


Figure 1b: Image of the Experimental layout in the RTG Lab

***
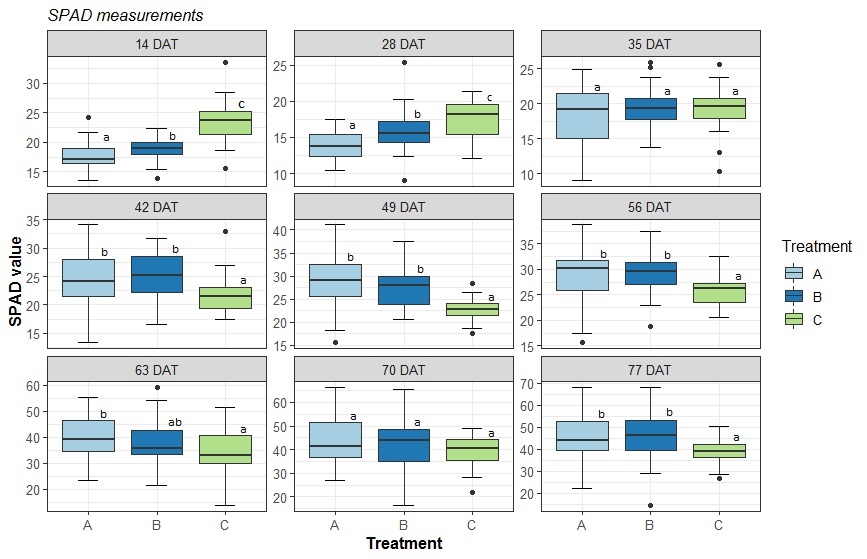
***

Figure 2b: Chlorophyll content measurements ( SPAD) in Phaseolus vulgaris leaves. Boxplot (n=32) results given for three treatments A) 2g of struvite + Rhizobium inoculation + P, Mg, N-free nutrient solution B) 5g of struvite + Rhizobium inoculation + P, Mg, N-free nutrient solution and C) standard nutrient solution - Rhizobium inoculation measured at 9 time periods throughout the crop cycle. Significant differences (p< 0.05) between treatments marked with different letter (a,b,c).

***
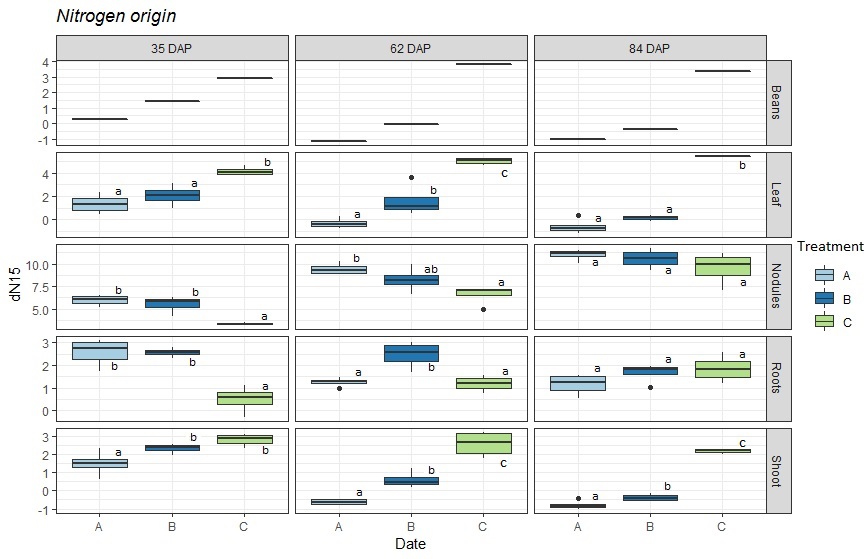
***

Figure 3b: Nutrient concentration in Phaseolus vulgaris leaves and shoots, expressed in mg/g. Boxplot (n=4) results given for three treatments A) 2g of struvite + Rhizobium inoculation + P, Mg, N-free nutrient solution B) 5g of struvite + Rhizobium inoculation + P, Mg, N-free nutrient solution and C) standard nutrient solution - Rhizobium inoculation at three different time periods. 35 days after transplanting, 62 days after transplanting and 84 days after transplanting. Significant differences (p< 0.05) between treatments marked with different letter (a,b,c).

***
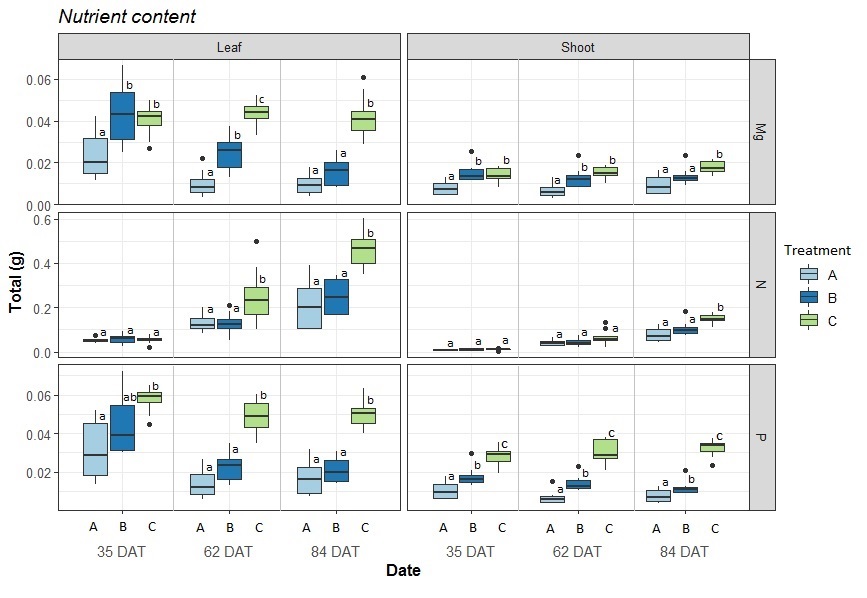
***

Figure 4b: Nutrient content in Phaseolus vulgaris leaves and shoots, expressed in g. Boxplot (n=4) results given for three treatments A) 2g of struvite + Rhizobium inoculation + P, Mg, N-free nutrient solution B) 5g of struvite + Rhizobium inoculation + P, Mg, N-free nutrient solution and C) standard nutrient solution - Rhizobium inoculation at three different time periods. 35 days after transplanting, 62 days after transplanting and 84 days after transplanting. Significant differences (p< 0.05) between treatments marked with different letter (a,b,c).


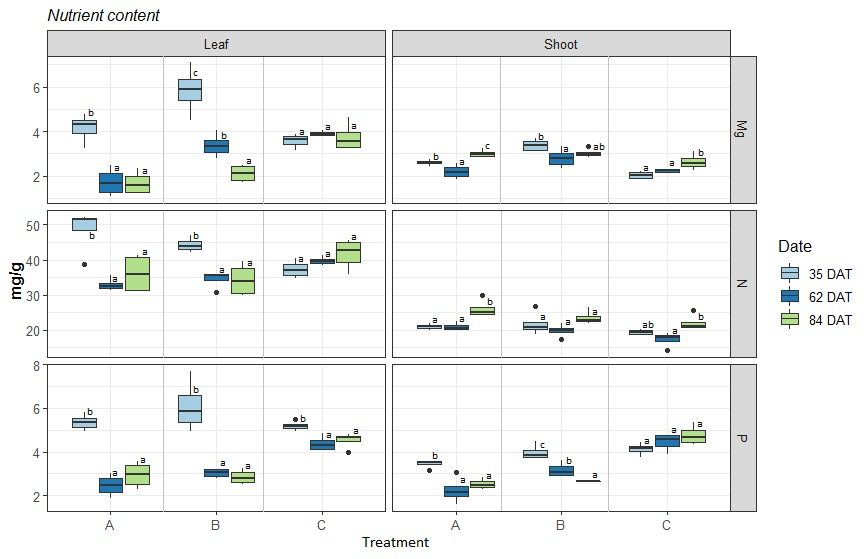


Figure 5b: Nutrient content in Phaseolus vulgaris leaves and shoots, expressed in mg/g. Boxplot (n=4) results given for three treatments A) 2g of struvite + Rhizobium inoculation + P, Mg, N-free nutrient solution B) 5g of struvite + Rhizobium inoculation + P, Mg, N-free nutrient solution and C) standard nutrient solution - Rhizobium inoculation at three different time periods. 35 days after transplanting, 62 days after transplanting and 84 days after transplanting. Significant differences (p< 0.05) between dates marked with different letter (a,b,c).
